# Cationic lipid-based nanoparticles for therapeutic delivery in cancer treatment: physicochemical characteristics, therapeutic cargos, and clinical potential

**DOI:** 10.1186/s42649-026-00130-0

**Published:** 2026-04-02

**Authors:** Chun-Sik Bae, Taeho Ahn

**Affiliations:** College of Veterinary Medicine, Chonnam National University, 77 Yongbong-ro, Buk-gu, Gwangju, 61186 Republic of Korea

**Keywords:** Cationic lipid-based nanoparticles, Cancer therapy, RNA interference, Protein and peptide delivery, Drug delivery platform

## Abstract

Over the past few decades, numerous biological therapeutics, such as RNAs and polypeptides, have emerged as promising alternatives to traditional chemotherapy. While their molecular mechanisms of action are well-understood, their clinical application remains hindered by several critical barriers, including inherent intracellular instability, the need for precise target-site delivery, poor cellular uptake, and immune system clearance. To overcome these challenges, the development of selective delivery systems has been raised as an indispensable strategy. Among various drug carriers, cationic lipid-based platforms have garnered significant attention and are increasingly exploited in oncology. This review summarizes the physicochemical characteristics of cationic lipid-based nanoparticles and evaluates various therapeutic cargos based on their biological properties. Furthermore, we explore the current research applications and clinical potential of these nanoparticles in cancer treatment.

## Introduction

Cancer is a leading cause of mortality globally in both humans and animals. Despite extensive studies and significant advancements in tumor therapies, substantial barriers persist regarding their pharmacological application. Conventional chemotherapy, which relies on small-molecule anti-cancer drugs, has been widely administered to the patients. However, these drugs typically lack specificity, frequently causing severe systemic toxicity (Allen 2002). Furthermore, the emergence of multidrug resistance (MDR) in cancer cells often necessitates the premature discontinuation of the treatment (Holohan et al. 2013).

To address these limitations, considerable attention has shifted toward highly potent macromolecular drugs, such as inhibitory RNA for gene silencing and pro-apoptotic proteins/peptides, which are regarded as promising anti-cancer candidates. Nevertheless, the clinical application of these “bio-drugs” faces several critical disadvantages. For small interfering RNAs (siRNA) and other inhibitory RNAs, the primary challenges involve inherent instability and the difficulty of efficient delivery to the target site. Additionally, siRNA can elicit off-target effects−silencing genes with sequences similar to the target−and trigger innate intracellular immune responses (Zhang et al. 2023a). Low cellular uptake remains another major barrier to the clinical and pharmaceutical exploitation of these macromolecules (Zhang et al. 2019).

Similarly, the clinical use of protein- and peptide-based drugs for cancer treatment is hampered by poor membrane permeability, low stability, susceptibility to proteolysis, immunogenicity, and rapid renal clearance (Cheshomi et al. 2026). Unlike small-molecule drugs, protein structures are highly sensitive to environmental factors such as temperature and pH, complicating the maintenance of conformational stability during manufacturing. Furthermore, tumors often exhibit high interstitial fluid pressure and a dense extracellular matrix. These physical barriers impede the penetration of RNA or protein-based therapeutics into the tumor core, potentially leaving the center untreated and leading to disease recurrence (Sheng et al. 2026). Consequently, the development of sophisticated drug formulation utilizing selective delivery systems has emerged as an indispensable strategy to mitigate these drawbacks.

While existing literature often focuses narrowly on individual nucleic acid carriers, this review provides a critical and integrated perspective on the evolutionary trajectory of cationic lipid-based nanoplatforms. By analyzing the structural shift from first-generation permanent cationic lipids to next-generation ionizable architectures, it is explored how these strategic advancements facilitate the transition from preclinical promise to clinical reality.

## Main text

### RNA interference: mechanism and therapeutic potential

RNA interference (RNAi) is a conserved biological process that silences gene expression by promoting the degradation of messenger RNA (mRNA) (Lam et al. 2015). This mechanism utilizes double-stranded endogenous or exogenous non-coding RNA molecules−such as siRNA, microRNAs (miRNAs), and short hairpin RNAs (shRNAs)−which are targeted to degrade mRNA in a sequence-specific manner (Lam et al. 2015; Sioud 2020). By inhibiting the production of proteins that drive oncogenesis, RNAi is currently being harnessed as a powerful precision tool for cancer therapy.

The RNA-induced gene silencing pathway begins when double-stranded RNA (dsRNA), upon crossing the cell membranes, is cleaved into smaller duplex RNA (siRNA) by the enzyme Dicer. These siRNAs are subsequently incorporated into the RNA-induced silencing complex (RISC). Within this complex, the endonuclease Argonaute 2 (Ago-2) unwinds the duplex into two single-stranded RNAs: the “passenger” (sense) strand, which is degraded or released, and the “guide” (antisense) strand, which directs the RISC to the complementary target mRNA sequence. Finally, the Ago-2 mediated complex cleaves the target mRNA thereby inhibiting mRNA translation and achieving the gene knockdown (Sioud 2020). Exogenous siRNA can function in the same manner as natural dsRNA; therefore, synthesized siRNAs are frequently utilized as cargo in delivery systems to suppress various cancer-related genes (e.g., *c*-Myc) (Chen et al. 2010).

In terms of functional mechanisms, siRNA typically induces highly specific gene silencing because the antisense strand binds only to mRNA with perfect complementarity. In contrast, miRNA−which originates in the nucleus and possesses a stem-loop structure−binds imperfectly to mRNA and can degrade multiple mRNA targets, potentially leading to off-target toxicity (Lam et al. 2015). Accordingly, RNAi therapy offers a versatile platform, allowing for the design of drug targeting virtually any disease-promoting gene based on its mRNA sequence (Hu et al. 2019). Furthermore, siRNA is often preferred over other gene therapies because it is easily synthesized and does not require genome integration, thereby avoiding risks of mutation and teratogenicity (Xu and Wang 2015). Additionally, siRNA delivery is more straightforward than that of shRNA plasmid or plasmid DNA because its site of action is the cytosol. Its small size, higher transfection efficacy, potency, and relatively lower immune response, make it an ideal candidate for RNAi therapeutics (Sajid et al. 2020).

Despite these advantages, the anionic nature and high molecular mass of siRNA present significant barriers to crossing negatively charged cell membranes (Brock et al. 2019). Following cellular entry, entrapped siRNA-carrier vesicles are transported from early endosomes to late endosomes (pH 5−6), and eventually to lysosomes (pH ~ 4.5), where they face degradation by nucleases. Consequently, siRNAs must escape into the cytosol (pH ~ 7.4) at an early stage; this endosomal escape is the rate-limiting step for efficient siRNA delivery (Bardoliwala et al. 2019; Brock et al. 2019).

To facilitate cellular uptake and endosomal escape of siRNA, effective delivery strategies are essential; in this context, cationic lipid-based nanoparticles (cLNPs) have garnered significant attention. The primary pathway for the non-viral delivery of siRNA is endocytosis. Generally, the cationic moiety in siRNA-carrier interacts with anionic proteoglycans on the cell surface to trigger internalization (Kasai et al. 2019). Clathrin-dependent endocytosis is the most well-characterized pathway for the nanocarrier-mediated siRNA uptake, although the optimal route varies by cellular context (Vocelle et al. 2020). Nanoparticles (NPs) smaller than 150 nm typically avoid macrophage uptake and ensure efficient endocytosis (Rejman et al. 2004). Alternatively, caveolae-dependent endocytosis may mediate the entry of larger complexes (approaching 500 nm), although this remains an ongoing discussion (Behzadi et al. 2017). To further enhance endosomal escape, various strategies have been investigated, including the use of fusogenic lipids, such as DOPE (1,2-dioleoyl-l-a-glycero-3 phosphatidylethanolamine), or pH-sensitive fusogenic peptides as a component in nanocarriers. These agents destabilize the endosomal membranes in low pH environments to release the cargo into the cytoplasm (Álvarez-Benedicto et al. 2022; Grau and Wagner 2024).

RNAi therapies are designed to disrupt multiple stages of cancer progression (John et al. 2025). They can directly target oncogenes that promote uncontrolled cell division, silence genes responsible for MDR (e.g., MDR1), or inhibit anti-apoptotic factors to resensitize tumors to standard treatment. Furthermore, they can block the expression of the vascular endothelial growth factors (VEGF) to inhibit tumor angiogenesis. Despite these multifaceted advantages, challenges persist regarding the inherent instability of naked RNA molecules, the requirement for tumor-specific targeting, and the minimization of off-target effects. Therefore, advancements in lipid nanoparticles (LNPs) and ligand-conjugated systems are being aggressively pursued (Ward et al. 2021). As of early 2026, while several RNAi therapies are U.S. Food and Drug Administration (FDA)-approved for rare genetic and metabolic diseases (e.g., Onpattro and Givlaari), most cancer-specific RNAi therapies remain in clinical trial phases (Traber and Yu 2024).

### Protein and peptide therapeutics

Protein therapeutics have emerged as a crucial strategy for treating cancer, immunological diseases, and metabolic disorders (Gu et al. 2011). Compared with chemotherapy and gene therapy, protein drugs offer unique advantages in cancer therapy (Mitragotri et al. 2014). First, they exhibit high potency and specificity by either directly inducing cancer cell apoptosis or indirectly inhibiting tumors through modulation of the tumor microenvironment (TME) or stimulation of the immune response (Scott et al. 2012). Due to these properties, protein drugs usually display significantly lower IC_50_ (half maximal inhibitory concentration) values than conventional chemotherapeutic agents and are less toxic to healthy tissues (He et al. 2016). Moreover, protein therapeutics are less genotoxic than gene therapy because they function via downstream regulation mechanisms that do not alter the host’s genetic makeup (Leader et al. 2008).

In addition to protein therapy, peptides with shorter amino acid sequences are also extensively explored and utilized due to their smaller sizes and ease of manipulation (Qi et al. 2018). However, delivering pharmacologically active proteins or peptides to specific tissues or cells encounters several challenges, including instability in blood circulation, enzymatic degradation, short half-lives, immunogenicity, and the inability to cross cell membranes (Liu et al. 2025). Consequently, various protein delivery systems have been developed to encapsulate these molecules, protecting them from denaturation and degradation, promoting tumor-targeted delivery, enhancing transmembrane efficiency, and providing controlled release at target sites (Zhu et al. 2024, 2025).

### Cytokines and antibodies

In cancer therapy, cytokines can directly induce tumor cell apoptosis or indirectly kill tumor cells by regulating immune responses (Yin et al. 2025). Various cytokines, including interleukins (e.g., IL-2 and IL-12), interferons (e.g., INF-γ), and tumor necrosis factors (e.g., TNF-α and TNF-β), have been applied in treatment. However, the clinical use of systemic cytokines at high doses is severely restricted by dose-limiting toxicities that can lead to multi-organ failure (Song 2024). These side effects are primarily driven by uncontrolled inflammatory cascades and vascular damage. To mitigate these risks, researchers are shifting from systemic bolus injections toward targeted delivery systems, such as immunocytokines (cytokines fused to antibodies) and NP encapsulation (Wang et al. 2025).

Therapeutic antibodies are among the most successful approaches for cancer treatment, eliciting selective anti-tumor effects by targeting oncogenic proteins or survival factors (e.g., HER2, EGFR, and VEGF) on cell surfaces (Paul et al. 2024). These antibodies block essential growth signals and tag cancer cells for destruction via mechanisms like antibody-dependent cellular cytotoxicity. Advanced strategies include antibody−drug conjugates, which deliver cytotoxic drugs directly to malignant cells, (Okpasuo et al. 2026), and immunotoxins, which link antibodies to potent bacterial or plant toxins like diphtheria toxin (Li et al. 2017). Unlike chemotherapy, a single toxin molecule can be lethal once internalized (Kim et al. 2022). However, these toxins often trigger neutralizing antibodies and capillary leak syndrome. Current research focuses on de-immunization−using protein engineering to remove B-cell and T-cell epitopes−to extend the therapeutic window (Mazor and Pastan 2020).

### Pro-apoptotic proteins and anti-cancer peptides

The pro-apoptotic proteins and peptides activate the caspase-dependent pathway or function as apoptosis-inducing factors (Dho et al. 2025; Chen et al. 2024). Cytochrome *c* (Cyt *c*) is a notable example; its release from the mitochondria induces the intrinsic apoptosis pathway (Jiang and Wang 2004). Because many cancer cells survive by blocking the release of Cyt *c* (e.g., via Bcl-2 overexpression), researchers use lipid or mesoporous silica NPs to deliver exogenous Cyt *c* directly into tumor cells, bypassing internal survival signals (Bae et al. 2020; Delinois et al. 2021). Co-delivering Cyt *c* from mitochondria apoptosis-inducing factor (AIF) may bypass drug resistance in tumors with defective caspase machinery (Lorenzo and Susin 2007). Other agents, such as tumor necrosis factor-related apoptosis-inducing ligand (TRAIL) and TRAIL-mimetic peptides are also being explored for their potent cell-death-inducing properties (Min et al. 2018).

Anti-cancer peptides (ACPs) serve as a highly selective alternative to traditional chemotherapy (Chinnadurai et al. 2023). Typically 5–50 amino acids long, ACPs exploit the negative surface charge and high membrane fluidity of cancer cells (Xie et al. 2020). They function by forming pores in cell membranes, targeting mitochondria, or inhibiting tumor blood vessel growth. Despite the FDA approval of drugs like Bortezomib and Tebentafusp, the clinical utility of many therapeutic peptides is limited by their rapid degradation (Zheng et al. 2025). To overcome this, NPs including liposomes are increasingly used as carriers to ensure these peptides reach the tumor site intact.

### Cargo-free nanoparticles

Beyond their general role as passive drug carriers, cargo-free or “self-therapeutic” NPs are emerging as potent anticancer agents in their own right. These systems exploit their unique physicochemical attributes to combat cancer cells and reprogram the TME. Specifically, cargo-free nanoparticles (CFNPs) are being explored for oncological applications due to the electrostatic attraction between their positive surface charge and the typically negative charge of cancer cell membranes (Vishwakarma et al. 2024). Consequently, these systems are often referred to as “intrinsic” NPs because of their inherent therapeutic properties (Wang et al. 2017).

CFNPs can be engineered to be cytotoxic to malignant cells without incorporating traditional chemotherapeutic or biological agents. For example, cationic guanidium-functionalized poly(l-lysine) is self-assembled into NPs that induce apoptosis in cancer cells (Yang et al. 2022). Recent research demonstrates that these particles exhibited efficacy comparable to drug-loaded versions in breast cancer models (e.g., MCF-7/ADR). Furthermore, CFNPs containing poly(dl-lactide-co-glycolide) have been shown to reprogram innate immune cell phenotypes, thereby altering the primary TME (Zhang et al. 2022a). This “immune reprogramming” strategy works by reversing local immunosuppression, enabling the host’s immune system to recognize and attack metastatic lesions. Additionally, CFNPs can deliver tumor-associated antigens directly to dendritic cells to elicit a robust antigen-specific cellular immune response (Heuts et al. 2021). Some CFNPs have also been used to redirect pro-tumor immune cells away from the TME to decelerate tumor growth (Parveen et al. 2025).

**Fig. 1:**
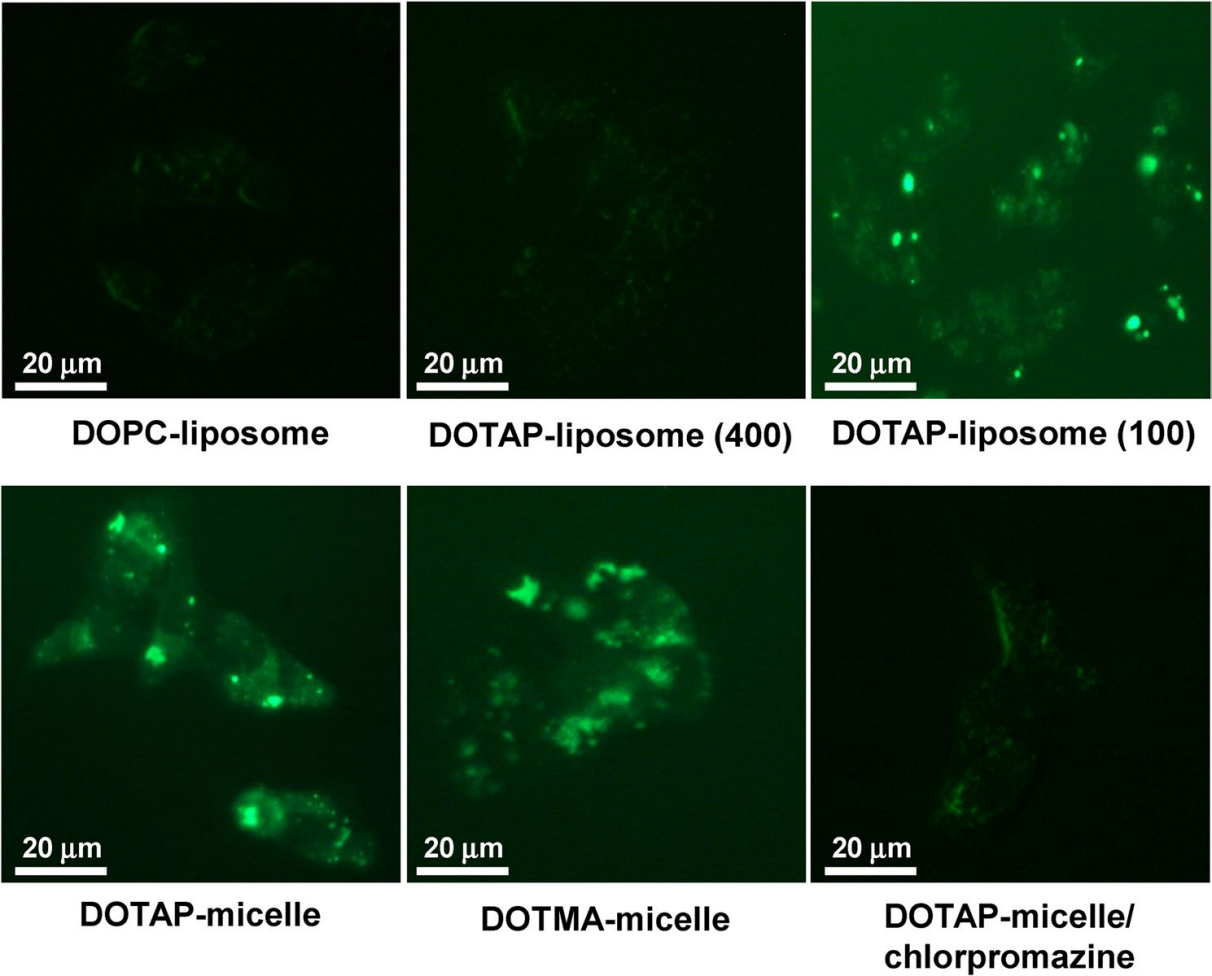
Uptake of NPs into HepG2 cells. The cellular uptake of NPs into cells was imaged using 18:1 1,2-dioleoyl-3-phosphoethanolamine-N-(carboxyfluorescein) incorporated into NPs under a fluorescence microscope. Chlorpromazine is a clathrin-mediated endocytosis inhibitor. The numbers in parentheses represent the diameter (nm) of DOTAP-liposomes. Adapted with permission from Bae et al. 2016

Beyond direct cytotoxicity, CFNPs can function as intrinsic adjuvants; their positive charge serves as a “danger signal” that enhances antigen presentation to dendritic cells and promotes T-cell infiltration into “cold” tumors (Heuts et al. 2021). CFNPs can also induce oxidative stress and subsequent cell death, with effects depending on the lipid concentration, surface charge density, lipid composition, and particle size (Bae and Ahn 2018; Yun et al. 2016) (Fig. 1). Some formulations, including specific silica CFNPs, are engineered to disrupt cell membranes or interfere with metastatic pathways directly (Tang et al. 2024; Esfahani et al. 2022). Even neutrally charged polypeptide NPs without cargo have demonstrated anti-cancer potential through their structural interactions with cancer cells that induce apoptosis (Yang et al. 2022). These drug-free particles offer several superiorities over conventional cargo-loaded carrier systems. They achieve maximum drug loading efficiency since the NPs themselves function as the pharmaceutical agent. Furthermore, reduced systemic toxicity (Mei et al. 2021), enhanced pharmacokinetics and accumulation (Zhou et al. 2013), and direct biological activity (Wang et al. 2018a) represent pivotal clinical benefits. These attributes, combined with streamlined synthesis and scalability (Zhang et al. 2023b), make CFNPs attractive for pharmacological applications.

However, many CFNPs face significant hurdles. Inorganic materials, such as non-biodegradable silica, frequently accumulate in the reticuloendothelial system (notably the liver and spleen), potentially causing chronic inflammation, oxidative stress, and cell death (Havelikar et al. 2024). CFNPs also struggle to penetrate dense TME, which is characterized by a rigid extracellular matrix with high interstitial fluid pressure, as these NPs lack a loaded drug to diffuse independently (Munir 2022). Without active targeting ligands or intracellular-targeting strategies, these NPs may exhibit poor cellular internalization, leading to suboptimal therapeutic outcomes (Paresishvili and Kakabadze 2023; Mei et al. 2021). Furthermore, the rapid formation of a “protein corona” in the bloodstream can signal the immune system to clear the particles before they reach the tumor. While many such systems show promise in animal models, translating these results to human trials remains difficult due to low bioavailability or a lack of superior efficacy compared to standard-of-care treatments. Despite these challenges, CFNPs are being rigorously tested−primarily in preclinical stages−as a sophisticated alternative to traditional drug-carrier complexes.

### Metals

Although they are not organic compounds and do not contain any biological payloads, metal-based nanoparticles (MNPs) represent one of the most versatile platforms in oncology due to their unique optical, magnetic, and catalytic properties. In addition, they are central to the field of theranostics, in which diagnosis and therapy are integrated into a single system (Ding et al. 2026). For instance, iron oxide NPs are utilized in magnetic hyperthermia for their superparamagnetic properties, where an alternating magnetic field generates heat to remove cancer cells (Garg et al. 2025). Silver NPs possess intrinsic anticancer properties, inducing oxidative stress within cancer cells and are frequently employed to enhance the efficacy of traditional chemotherapy (Rivera et al. 2024). Additionally, gold NPs are highly stable and biocompatible; they are primarily used in photothermal therapy to convert near-infrared light into heat for the thermal ablation of tumors (Baker et al. 2023). To improve targeting and minimize systemic toxicity−as metals can cause long-term organ toxicity if not cleared efficiently−MNPs can be functionalized with ligands, such as antibodies and peptides, that bind to specific receptors overexpressed on cancer cells (Gutiérrez et al. 2023).

### Viral and non-viral vectors

Viral vectors are a prominent tool for siRNA delivery; they typically contain shRNA, which is subsequently processed by the cell into siRNA to silence specific genes (Ghasemiyeh and Mohammadi-Samani 2025). Given their inherent transduction efficiency, viral vectors deliver genetic material directly into the host cell, often surpassing the performance of non-viral alternatives. Currently, Adenoviruses, adeno-associated viruses (AAV), and lentiviruses currently account for over 80% of approved viral-based therapies. In addition, many viral vectors have been engineered with specific proteins, such as tumor-specific markers, to target particular cell types (tropism); this precision reduces systemic toxicity and unintended “off-target” effects in healthy tissues (He et al. 2024).

Leveraging these properties, viral vectors are utilized to deliver siRNA into difficult-to-reach cells, including non-dividing cells like neurons−which are often resistant to chemical transduction (Ali Zaidi et al. 2023). By ablating natural tropism, higher doses can be administered with fewer side effects. Current in vivo applications of viral vectors are shifting toward direct injection into the bloodstream rather than complex ex vivo cell modification (Andorko et al. 2025). For example, clinical trials are now utilizing engineered lentiviral or AAV vectors to target T cells within the bloodstream, transforming them into chimeric antigen receptor (CAR)-T cells without the need for specialized manufacturing facilities (Xu et al. 2025). Advanced viral vectors are also being designed with specific envelopes or capsids to ensure they infect only the intended immune cells or tumor sites, thereby bypassing healthy organs. However, the body’s immune system may recognize viral proteins, potentially triggering inflammatory responses or neutralizing the vector before it reaches its target. Additionally, viral vectors have physical limits on the size of genetic cargo that they can carry, and producing clinical-grade viral vectors remains expensive, technically demanding, and less scalable than synthetic alternatives like LNPs (Ren et al. 2025).

For applications prioritizing safety and ease of use, non-viral delivery systems, such as LNPs, are increasingly favored for transient siRNA delivery and other bio-drugs. In contrast, viral vectors remain the “gold standard” for high-efficiency and long-term applications (Geng et al. 2025). Current non-viral platforms applied for the drug delivery include lipid-, polymer-, peptide-based delivery systems, as well as hybrid NPs (Wang et al. 2023). While non-viral carriers generally lack the high level of tissue tropism and transfection efficiency compared to their viral counterparts (Al-Dosari and Gao 2009), they are widely pursued due to their relative safety, low-cost production, and ability to evade the immune system (Ren et al. 2021).

### Cationic lipid-based delivery systems

**Fig. 2:**
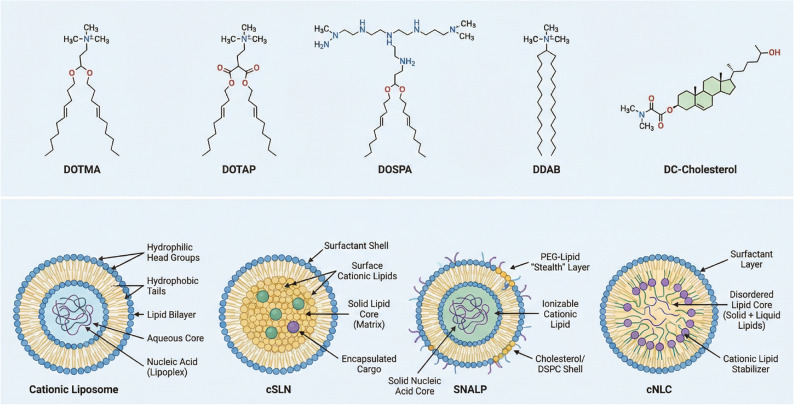
Chemical structures of cationic lipids and schematic representations of cationic-lipid based nanoparticles for nucleic acid delivery. Chemical structures of representative cationic lipids used in gene delivery systems, including DOTMA, DOTAP, DOSPA, DDAB, and DC-Cholesterol. Schematic illustration of four major cLNP platforms: Cationic liposome featuring a lipid bilayer and aqueous core; cSLN (Cationic Solid Lipid Nanoparticle) consisting of a solid lipid matrix and surfactant shell; SNALP (Stable Nucleic Acid Lipid Particle) characterized by a PEG-lipid "stealth" layer and ionizable cationic lipids; and cNLC (Cationic Nanostructured Lipid Carrier) composed of a disordered core of mixed solid and liquid lipids

Cationic lipid-based systems are among the most extensively investigated platforms for siRNA delivery. These include the forms of cationic liposomes, cationic solid lipid nanoparticles (cSLNs), stable nucleic acid lipid particles (SNALPs), and cationic nanostructured lipid carriers (cNLCs), which are collectively referred to as cationic nanoparticles (cNPs) (Vishwakarma et al. 2024). cNPs are widely used to deliver nucleic acid into target cells due to their multivalent cationic charges, facilitating robust electrostatic interaction with polyanionic nucleic acids (Wang et al. 2015) (Fig. 2).

cNPs are formulated with cationic lipids, including monovalent N(1-[2,3-dioleyloxy]propyl)-N, N,N-trimethylammonium chloride (DOTMA), 1,2-dioleyl-3-trimethylammonium-propane (DOTAP), dioctadecylamidoglycylspermine, 2,3-dioleyloxy-N-[2-(sperminecarboxamido)ethyl]-N, N-dimethyl-1-propanaminium (DOSPA), and dimethyldioctadecylammonium bromide (DDAB), as well as multivalent lipids like 3β-(N-[N’,N’-dimethylaminoethane]-carbamoyl)cholesterol (DC-Cholesterol) (Podesta and Kostarelos 2009). These lipids can be used in isolation or mixed with other lipids and polymers. Among these, DOTAP and DOTMA−often termed “first generation” cationic lipids−remain prominent in siRNA and drug delivery research due to several distinct advantages over other cationic lipids. They maintain a permanent positive charge across all physiological pH levels, which simplifies NP self-assembly and ensures stable electrostatic interaction with siRNA and mRNA. Furthermore, their strong positive charge promotes rapid interaction with anionic cellular membranes, enhancing cellular uptake and facilitating efficient endosomal escape through membrane destabilization. Consequently, these lipids can transfect a broad range of cell lines, including challenging targets such as immune cells and non-dividing cells (Lamparelli et al. 2025).

In oncology, research indicates that DOTAP- and DOTMA-based NPs effectively activate innate immunity and induce potent T-cell responses (Chilumula et al. 2025). Beyond nucleic acid delivery, DOTAP has been employed for polypeptide antigen delivery in cancer vaccines, where it serves as an adjuvant to bolster cell-mediated immunity (Vasievich et al. 2012). Given the importance of polypeptide-DOTAP systems in cancer therapies, various specialized formulations have been developed (Guo and Jiang 2020). Mechanistically, E7 peptide (EPLQLKM)-loaded DOTAP NPs have been shown to function as cancer vaccines by inducing the production of reactive oxygen species (ROS) in bone marrow-derived dendritic cells (Yan et al. 2008). Similarly, DOTMA facilitates effective siRNA delivery when complexed with cationic polymers (such as polyethylenimine) or receptor-targeted peptides, forming binary and ternary complexes (Tagalakis et al. 2011). DOTMA-containing particles are also utilized as lipo(poly)plexes or polyplexes for gene delivery (Welser et al. 2013). These DOTMA-NPs are recognized as highly promising non-viral vectors and adjuvants for stimulating immune responses in carcinoma treatment (Kawai et al. 2024).

### Cationic liposomes

As of 2025, liposomes are the only nanomedicine platform approved by the FDA for inhalation (Zhang et al. 2025). They serve as a cornerstone of siRNA delivery research, particularly for extrahepatic targets such as the lungs. While broadly categorized by their surface charge and loading methodology, two types of liposomes−cationic liposomes formed by complexation (lipoplexes) and neutral liposomes formed by encapsulation−have garnered significant attention for the delivery of siRNA and other biomolecular cargos (Lechanteur et al. 2018; Fan et al. 2025).

Cationic liposomes are now considered the most attractive nanocarriers for siRNA delivery. Typically, the positively charged head of cationic lipids binds with the anionic phosphate groups of the nucleic acid through spontaneous electrostatic interaction, forming lipoplexes with diameters of 50–200 nm (Sun and Lu 2023). Lipoplexes are characterized by their ease of preparation, ability to protect siRNA from degradation, and high transfection efficiency (Khatri et al. 2014). While cationic lipids possess the ability to directly fuse with anionic membranes and release their payload into the cytosol, lipoplexes are predominantly internalized via clathrin-mediated endocytosis (Rejman et al. 2005). Subsequently, the positive charge of cationic lipids increases within the acidic environment of endosome (including lysosome), augmenting the affinity between the lipoplex and the anionic endosomal membrane. This lipid exchange, facilitated by the fusogenic nature of the liposomes, promotes the release of siRNA into the cytosol (Pengnam et al. 2022). The transfection efficiency can be further optimized by adjusting the lipid: siRNA (Nitrogen to phosphate ratio) to achieve a slightly positive surface charge (Tseng et al. 2009).

Various modifications have been developed to shield cationic liposomes from non-specific interactions, reduce immunological responses and cytotoxicity, and enhance endosomal escape. A primary strategy involves surface modification with polyethylene glycol (PEG) to create “stealth liposomes” (Abdel-Mageed et al. 2021). While PEGylation is generally more advantageous for systemic delivery by evading the reticuloendothelial system, it can also improve transfection efficiency in pulmonary delivery (Nguyen et al. 2008). Thus, the optimal effect of PEGylation depends heavily on the specific delivery route and the therapeutic objectives. Furthermore, the inclusion of fusogenic lipids, such as DOPE, in cationic liposomes is thought to increase interactions between the liposomal and endosomal membranes, facilitating siRNA release (Mendonça et al. 2023). Several commercially available transfection agents, such as Lipofectin (DOTMA/DOPE) and Lipofectamine (DOSPA/DOPE), utilize these cationic lipid frameworks for siRNA transfection (Babu et al. 2016).

Despite the advantages of cationic liposomes−such as efficient in vitro transfection, high loading capacity, structural flexibility, biocompatibility, biodegradability, and scalability−their clinical applications are hindered by significant drawbacks. These include safety concerns, non-specific tissue uptake, and premature siRNA release resulting from interaction with proteins, lipoproteins, and the extracellular matrix (Ochoa-Sánchez et al. 2024). Furthermore, cationic liposomes are highly toxic to immune cells and can induce their clearance by macrophages via the complement cascade (Inglut et al. 2020). Unlike their neutral and negatively charged counterparts, they may induce lung inflammation and toxicity by stimulating the production of ROS (Dokka et al. 2000). Beyond size and charge, the toxicity of cationic liposomes are influenced by the concentration, the lipids: siRNA ratio, and the chemical nature of the lipids (Lechanteur et al. 2018). In terms of chemical structure, monovalent cationic lipids like DOTAP, are generally less toxic than multivalent counterparts like Lipofectamine due to their simpler structure and lower charge density, albeit at the cost of lower RNA-binding efficiency (Ojha et al. 2025).

Lipoplex-based protein and peptide delivery has emerged as a strategy to overcome the fragility of these molecules by leveraging the same electrostatic mechanisms used in gene therapy. Traditionally employed for nucleic acids, lipoplexes are being repurposed to protect proteins from enzymatic degradation and enhance their cellular uptake (Yu et al. 2016). Similar to nucleic acid binding, cationic lipids (e.g., DOTAP and DDAB) form complexes with anionic proteins via electrostatic attraction (Ochoa-Sánchez et al. 2024). For proteins that are not naturally anionic, fusion proteins−such as engineered human proteins or anionic Green Fluorescence Protein (GFP)−are used to impart a negative charge, enabling encapsulation by cationic lipids (Kim et al. 2019). The resulting lipoplexes facilitate delivery by destabilizing endosomal membranes after cellular uptake and releasing the protein cargo into the cytosol. As with nucleic acids, lipoplex encapsulation extends the systemic circulation half-life of proteins, potentially reducing the frequency of administration (Koide et al. 2022). Furthermore, lipoplexes provide a protective environment that prevents premature denaturation of unstable proteins (Koide et al. 2022). Current research is expanding into the co-delivery of proteins with other agents, such as chemotherapeutics (e.g., paclitaxel) or nucleic acids (siRNA/miRNA), to create synergistic therapeutic effects in cancer treatment (Al Bostami et al. 2022). This co-delivery approach represents a major frontier in overcoming drug resistance of cancer and enhancing therapeutic precision through multifaceted attacks on tumor cells.

### Cationic solid lipid nanoparticles

cSLNs are advanced drug delivery systems representing a critical intersection between traditional solid nanoparticles (SLNs) and the ionizable LNPs technology popularized by mRNA vaccines. They have been engineered to provide a stable and highly charged platform for the delivery of genetic medicines−such as DNA, siRNA, and mRNA−as well as hydrophobic drugs for cancer therapy (Gupta et al. 2025). Regarding cargo complexation, cSLNs have demonstrated a unique capacity to co-deliver Paclitaxel, a lipophilic chemotherapy drug, alongside siRNA (Yu et al. 2012). This co-delivery strategy has been refined to synergistically enhance antitumor effects and circumvent MDR of cancer cells (Creixell and Peppas 2012). Currently, cSLNs are increasingly utilized in gene therapy and vaccine development due to their high structural stability and ability to penetrate biological membranes (Llaguno-Munive et al. 2024).

A cSLN typically consists of three primary components: a solid lipid core, cationic lipids, and surfactants (or emulsifiers) (Giri et al. 2023). The solid lipid core is composed of biocompatible lipids, such as triglyceride (e.g., tristearin), fatty acids (e.g., stearic acid), or waxes. These lipids remain rigid solid at both room and body temperature, providing a stable matrix for drug encapsulation. Cationic lipids, such as DOTAP, DOTMA, or cetyltrimethylammonium bromide (CTAB) are integrated into the lipid matrix or onto the surface to provide a zeta potential typically ranging from +25 to +44 mV. This positive charge facilitates electrostatic binding with negatively charged nucleic acids. Surfactants are employed to stabilize the particles in aqueous dispersion and prevent aggregation. When formulating cSLNs, the choice of surfactant is critical, as it dictates particle size, physical stability, and biocompatibility (Botto et al. 2017). They are generally categorized into three groups based on their charge: non-ionic (e.g., Poloxamers), amphoteric (e.g., phospholipids), and ionic (e.g., SDS, CTAB, or DDAB) surfactants. Because each surfactant class presents specific advantages and disadvantages, mixtures−such as a combination of a non-ionic and an ionic surfactant−are frequently utilized. Additionally, helper lipids or modifiers, such as cholesterol or PEG-lipids, may be added to improve stability, extend circulation time, and reduce toxicity (Aldosari et al. 2021). For genetic agent delivery, the surface positive charge enables the formation of lipoplexes where nucleic acids are adsorbed or encapsulated, shielding them from enzymatic degradation in cells.

Compared to other lipid-based systems, cSLNs offer several unique benefits. To address the structural flexibility and potential for premature cargo leakage observed in traditional liposomes, cSLNs were engineered with a rigid solid lipid matrix (Viegas et al. 2023). This stable framework ensures the therapeutic payload remains intact until it reaches the target site, while also facilitating a sustained-release profile. Moreover, the core protects active ingredients from chemical degradation and enzymatic breakdown. The positive surface charge naturally interacts with the negatively charged membranes of cancer cells, promoting internalization more effectively than neutral or negative particles. Notably, cSLNs can cross the blood-brain barrier without compromising its integrity, making them promising candidates for treating central nerve system disorders (Qiao et al. 2024).

Despite their potential, the clinical application of cSLNs faces several critical challenges: Upon entering the bloodstream, they interact with serum proteins to form a “protein corona”. This interaction significantly alters their chemical composition, leading to rapid clearance by the mononuclear phagocyte system before they reach the tumor. This coating can also mask targeting ligands (e.g., antibodies or peptides) on cSLNs, rendering them ineffective against metastatic cell receptors (Kim et al. 2023). Furthermore, high positive surface charges can cause oxidative stress, activate pro-inflammatory signaling cascades, and disrupt cell membranes. Recent studies have noted that even biocompatible formulations may trigger transitory inflammation, such as increased macrophage populations in the lungs and liver following intravenous injection (Jacob et al. 2025). Due to their rigid crystalline structure, cSLNs inherently possess less space for cargo molecules than other lipid nanocarriers, resulting in lower drug-loading capacity. Additionally, the passive delivery of nanocarriers without active targeting strategies relies solely on the enhanced permeability and retention (EPR) effect, where NPs leak through porous tumor vasculature (Wu 2021). However, metastatic sites often lack these leaky vessels, hindering accumulation of nanocarriers. Consequently, many cSLN formulations struggle to maintain high specificity for metastatic tumor cells, complicating their clinical translation (Sivadasan et al. 2023).

To address these issues, research has focused on PEGylation to extend circulation time and the development of pH-responsive lipids that release their cargo only in specific environments (Chen et al. 2016a). To achieve the active targeting, current studies are functionalizing cSLN surfaces with ligands that recognize receptors overexpressed specifically on metastatic cells (Ying et al. 2022; Spada and Gerber-Lemaire 2025). Given the cytotoxicity associated with permanent cationic charges, a primary future perspective for cSLNs is the transition to ionizable lipids. These lipids remain neutral in systemic circulation−reducing toxicity and “protein corona” formation−but become positively charged within the acidic TME or endosomes to trigger cargo release. This strategy is currently being applied across almost all cLNP platforms in oncology. Researchers are also designing cSLNs capable of the simultaneous delivery of a hydrophobic chemotherapy drug (such as Paclitaxel) and a genetic agent. This co-delivery approach provides a synergistic attack on tumors, effectively overcoming MDR (Yu et al. 2012).

### Stable nucleic acid lipid particles

Originally developed to overcome the limitations of traditional lipoplexes, SNALPs evolved from stabilized antisense-lipid particle technology, which was initially designed to deliver antisense oligonucleotide (Semple et al. 2001). Zimmermann et al. (2006) provided the first definitive evidence that systemic RNAi could be achieved in non-human primates using SNALPs. They are currently recognized as the pioneering platform for systemic siRNA delivery with high efficiency and low toxicity in human clinical trials (Nele et al. 2024). These particles typically consist of four key components, a formulation strategy that now represents “gold standard” for siRNA delivery (Coutinho et al. 2020): Ionizable cationic lipids (e.g., DODMA or DODAP) remain neutral at physiological pH to reduce systemic toxicity but becomes positively charged in the acidic endosomal environment to facilitate siRNA release. PEGylated lipids provides a “stealth” layer that prevents particle aggregation and immune recognition, ensuring an extended circulation half-life in the body. Neutral helper lipids (e.g., DSPC) support the stable bilayer structure and cholesterol serves as a structural stabilizer, preventing cargo leakage from lipid matrix.

The SNALP platform achieved its most significant clinical milestone with the 2018 FDA approval of Onpattro (Patisiran), the first ever RNAi therapeutic. While SNALPs were initially developed for liver-based genetic diseases, their applications in oncology have expanded remarkably: They are now used to deliver siRNA that degrade mRNA of cancer-driving genes, such as PLK1 or KRAS (Pecot et al. 2014). Recent studies also suggest that SNALPs can simultaneously deliver siRNA for immune checkpoint knockdown (e.g., PD-L1) and mRNA for immune-stimulatory expression (e.g., OX40L) within the TME (Walters et al. 2021). Furthermore, they are employed to target MDR genes, thereby sensitizing resistant tumors to conventional chemotherapy. Beyond siRNA, this architecture provides the foundation for mRNA vaccines−including those for COVID-19 and emerging 2026 cancer vaccines−as the lipid-based envelopes safely shield nucleic acids in the bloodstream and release them inside cells (Hou et al. 2021). The spontaneous complexation characteristic of early lipoplexes has largely been replaced by microfluidic mixing, enabling precise, large-scale production of SNALPs with uniform sizes (typically around 60–100 nm).

Despite establishing the precedent for LNP technology, the translation of SNALPs into effective cancer therapies faces physiological and immunological hurdles. A primary challenge is endosomal escape, a hurdle common to nearly all nanocarriers; it is estimated that less than 2% of the delivered nucleic acid successfully escapes the endosomal compartment to reach the cytoplasm (Gilleron et al. 2013). Additionally, while PEGylation enhances stability and circulation time, repeated administration can induce the production of anti-PEG antibodies. This may trigger the accelerated blood clearance phenomenon, leading to rapid elimination of the particles from the body (Fu et al. 2024). As with other NP systems, limited tumor penetration and off-target effects remain significant burdens for the pharmaceutical applications of SNALPs.

The developmental trajectory of SNALPs parallels that of other nanocarrier classes (Abaza et al. 2025; Kon et al. 2023). Current research is shifting toward advanced ionizable lipids and next-generation SNALPs with surface ligands−such as antibodies or aptamers−to prioritize precision targeting over passive accumulation. Moreover, SNALPs are increasingly investigated in combination with immunotherapies, such as checkpoint inhibitors, to modulate the TME and enhance the anti-tumor immune response. Consequently, the SNALP platform is recognized as the critical evolutionary link between liposomes and contemporary LNPs, providing the foundational architecture for the first generation of systemic RNA therapeutics and the mRNA vaccines.

### Cationic nanostructured lipid carriers

To address the inherent drug expulsion issues of crystalline SLNs, “second-generation” cNLCs have emerged as a more robust alternative. By deliberately mixing solid and liquid lipids to create a disordered internal structure, cNLCs maximize loading capacity and provide more internal space for complex biological cargos (Alkhamach et al. 2025). Concerning cNLCs, the incorporation of positively charged lipids into carriers can exploit electrochemical interactions to improve drug delivery and cellular uptake (Tucak-Smajić et al. 2023).

As cancer cells often exhibit a higher negative surface charge compared to healthy cells, cNLCs leverage this to enhance internalization through electrostatic attraction (Tucak-Smajić et al. 2023). Regarding the interaction with cargos, cationic lipids (e.g., oleylamine) in cNLCs allow for the spontaneous encapsulation and protection of negatively charged genetic materials, such as miRNA, siRNA, and mRNA. Moreover, by co-delivering traditional chemotherapeutic agents (e.g., Paclitaxel) with gene-silencing molecules, cNLCs can help overcome MDR mechanisms often found in refractory tumors (Creixell and Peppas 2012). As with other NPs, current formulations of cNLCs are designed to adjust their charge based on environmental pH, remaining stable in the bloodstream (~ pH 7.4) and releasing their cargo efficiently once inside acidic endosomes (pH 5.5−6.5) (Lu and Sun 2025). In clinical and research applications, cNLCs have been widely used for delivering miR-107 and miRNA-27a in models for squamous cell carcinoma and other solid tumors to inhibit cell migration and proliferation (Piao et al. 2012). cNLCs can be also surface-modified with ligands, such as hyaluronic acid or transferrin to specifically target receptor (e.g., CD44) overexpressed on tumor cells (Yang et al. 2013). Additionally, combining cNLCs with PEGylation represents a powerhouse strategy for advanced drug delivery. While cNLCs improve drug loading by mixing solid and liquid lipids, adding a PEG coating transforms them into “stealth” vehicles capable of navigating the body’s immune defenses. Based on these properties, cNLCs have become a pivotal tool in cancer treatment, primarily as high-efficiency, non-viral vectors for gene and combination therapy.

While cNLCs exhibit significant potential in cancer therapy, several key challenges must be addressed prior to clinical application (Wang et al. 2018b): High concentrations of cationic lipids can induce pro-apoptotic and pro-inflammatory cascades within cells and cause membrane irritation, ultimately leading to cell lysis. In addition, similar to other nanocarriers, cNLCs are prone to immune clearance; they can be removed from the bloodstream by mononuclear phagocytes, which limits their ability to reach the tumor site effectively. Achieving precise targeting is also a substantial hurdle. While the EPR effect facilitates passive accumulation in tumors, it is insufficient to prevent non-specific distribution and associated systemic side effects. Additionally, ensuring long-term physical stability during large-scale manufacturing and storage, while preventing the premature leakage of encapsulated drugs or genetic material, remains a significant formulation challenge.

Despite these obstacles, ongoing research focuses on optimizing cNLC formulation to balance delivery efficiency with biocompatibility. A primary objective is to minimize systemic side effects, such as cytotoxicity often associated with cationic surfactants (Alkhamach et al. 2025). Consequently, next-generation cNLCs increasingly incorporate biodegradable or biocompatible lipids and surface-engineered coating to enhance their safety profiles. To achieve active targeting, recent advancements emphasize ligand-functionalized cNLCs, which improve selectivity for specific malignancies (Rizwanullah et al. 2021). Furthermore, the integration of diagnostic agents into these carriers enables the real-time monitoring of drug distribution and therapeutic response, facilitating a transition toward precision medicine (Rizwanullah et al. 2021). The distinct physicochemical architectures, therapeutic advantages, and clinical considerations of the various cationic lipid-based nanoplatforms are systematically summarized and compared in Table 1.

**Table 1:** Comparative analysis of nanoparticle platforms for cancer therapy

Platform Type	Physicochemical Structure and Characteristics	Key Advantages	Typical Size & Clinical Considerations
Cationic Liposomes	Composed of a lipid bilayer surrounding an aqueous core	High structural flexibility, biocompatibility, and ease of preparation	50–200 nm; prone to nonspecific tissue uptake and potential systemic toxicity due to high charge density
Cationic Solid Lipid Nanoparticles (cSLNs)	Features a rigid solid lipid matrix core stabilized by surfactants	Prevents drug leakage, facilitates sustained release, and can cross the blood-brain barrier	50−1,000 nm; lower loading capacity due to crystalline structure; potential for rapid clearance by the immune system
SNALPs (Ionizable LNPs)	Characterized by ionizable cationic lipids and a PEG-lipid “stealth” layer	The “gold standard” for systemic siRNA delivery; remains neutral in circulation to reduce toxicity	60–100 nm; FDA-approved (e.g., Onpattro) but may trigger anti-PEG antibodies upon repeated use
Cationic Nanostructured Lipid Carriers (cNLCs)	A “second-generation” platform with a disordered core of mixed solid and liquid lipids	Maximizes drug loading capacity and prevents cargo expulsion by creating more internal space	50−1,000 nm; leverages electrochemical interactions for enhanced uptake but faces challenges in precise targeting
Cargo-free Nanoparticles (CFNPs)	“Self-therapeutic” particles that do not carry exogenous drugs	Leverages inherent physical/chemical properties(e.g., positive charge) to induce apoptosis or modulate the TME	Achieves maximum loading efficiency(as the NP is the drug); however, inorganic versions may cause long-term organ toxicity
Metal-based Nanoparticles (MNPs)	Inorganic platforms with unique optical, magnetic, and catalytic properties	Ideal for theranostics(integrated diagnosis and therapy) and photothermal/magnetic hyperthermia	Highly stable but requires functionalization to minimize systemic organ toxicity from heavy metals

### Challenges and perspectives

Although cLNPs have shown significant promise in cancer research, their clinical translation remains constrained by several critical challenges. Primary barriers include the biological complexity and heterogeneity of tumors, as well as the limited penetration of NPs into dense solid tumors (Younas et al. 2025; Shin et al. 2026). Furthermore, concerns about long-term biocompatibility and potential off-target effects are unresolved hurdles to their widespread clinical application. Another notable challenge is the inherent toxicity associated with high positive charges, which can damage cell membranes and induce hemolysis (Fröhlich 2012). Upon intravenous injection, these NPs often form large complexes with serum proteins, known as a “protein corona”, leading to potential capillary blockade, thrombotic events, or rapid clearance by the mononuclear phagocyte system (Mayordomo et al. 2025). Achieving deep penetration into solid tumors is also difficult, as these particles tend to bind to the first cells they encounter at the tumor periphery (Miao et al. 2016). Regarding manufacturing, scaling up the synthesis of complex, multi-functional cationic carriers while maintaining consistent batch-to-batch physicochemical properties remains a significant hurdle for FDA approval (Li et al. 2025).

The clinical translation of cLNPs is no longer limited by cargo protection alone, but by the complex interplay between the nanoparticle surface and the heterogeneous TME. To transcend the limitations of the passive EPR effect, contemporary research is shifting toward “smart” functionalization−utilizing active targeting ligands and biomimetic coatings derived from cell membranes to achieve deep intratumoral penetration and avoid immune clearance. To reduce the systemic toxicity of cationic NPs, the most effective strategy is to mask their charge during systemic circulation. Coating NPs with PEG creates a hydration layer that shields the positive charge, thereby preventing non-specific binding with blood proteins such as opsonins (e.g., IgG and C3b) and extending circulation half-life (Mehta et al. 2023). To avoid immune clearance and improve biocompatibility, cationic cores can be encapsulated in membranes derived from erythrocytes, platelets, or even cancer cells; these biomimetic coatings allow NPs to bypass the immune system and utilize natural homing mechanisms to target tumors. To facilitate deep intratumoral penetration, surface modification with peptides such as iRGD (cyclic CRGDKGPDC) can trigger transcytosis, enabling the NPs to traverse endothelial layers and reach deep tumor tissues (Kang et al. 1906). To minimize off-target effects, “smart” NPs are designed to release their payload only in response to specific internal triggers, such as pH changes, enzymes (e.g., matrix metalloproteinases), or the redox potential within tumor cells (Zhang et al. 2022b). External triggers−including light (photothermal therapy), heat, or ultrasound−have also been explored to enable precise spatiotemporal drug release. While cationic charges in the NPs provide generalized “sticky” binding, the addition of active targeting ligands (e.g., antibodies, aptamers, or folic acid) ensures that the NPs bind preferentially to receptors overexpressed on malignant cells. This high selectivity reduces the required dose and limits collateral damage to healthy tissue (Chehelgerdi et al. 2023).

cNPs are increasingly recognized as effective vehicles for delivering tumor-associated antigens (mRNA, DNA, peptides) to dendritic cells, where they act as inherent adjuvants to stimulate potent T-cell responses (Gurunathan et al. 2024). Current developments are shifting toward “shielded” particles (such as PEGylated or mRNA-complexed formulation) that only expose their cationic charge upon reaching the acidic TME or in response to specific triggers. Furthermore, contemporary studies are focusing on the integration of cationic carriers with photothermal therapy and immune checkpoint inhibitors to prevent metastasis and recurrence (Shang et al. 2025; Chen et al. 2016b). As precision nanomedicine evolves to tailor NP characteristics to a patient′s unique tumor biology, the adoption of “green” synthesis methods of cNPs is becoming essential for sustainable development (Zhang et al. 2023c).

The future of cLNPs in oncology will be defined by their evolution into sophisticated theranostic platforms that seamlessly integrate therapeutic delivery with real-time diagnostic monitoring (Li et al. 2019). A critical frontier in this field is the adoption of multimodal hybrid imaging systems, such as PET/MRI and CT/SPECT, which overcome the limitations of individual modalities by combining high molecular sensitivity with superior anatomical resolution (Musafargani et al. 2018). These systems allow researchers to simultaneously track the biodistribution, pharmacokinetics, and therapeutic efficacy of cLNPs across multiple spatial and temporal scales (Saladino et al. 2025). Furthermore, emerging technologies like photoacoustic imaging and super-resolution microscopy are set to revolutionize our understanding of cLNP behavior at the nanoscale, providing unprecedented insights into intracellular trafficking, endosomal escape, and receptor-mediated uptake (Pramanik et al. 2022).

To manage the massive and complex datasets generated by these advanced imaging techniques, the integration of artificial intelligence (AI) and machine learning will become indispensable. AI-driven models can automate nanoparticle tracking, predict in vivo behavior based on physicochemical characteristics, and refine drug delivery precision, thereby streamlining the path toward clinical translation (Khakpour et al. 2025). However, significant regulatory and ethical challenges remain, including the high cost of infrastructure, the need for rigorous safety evaluations of new contrast agents, and concerns regarding data privacy in AI-enhanced diagnostics (Chamouni et al. 2025). Future efforts must prioritize the development of biocompatible and biodegradable imaging probes and foster global collaboration to ensure that these high-precision nanomedicines are accessible to diverse patient populations. This computational synergy, combined with multimodal theranostic capabilities, will define the next generation of personalized precision oncology.

## Conclusions

**Fig. 3:**
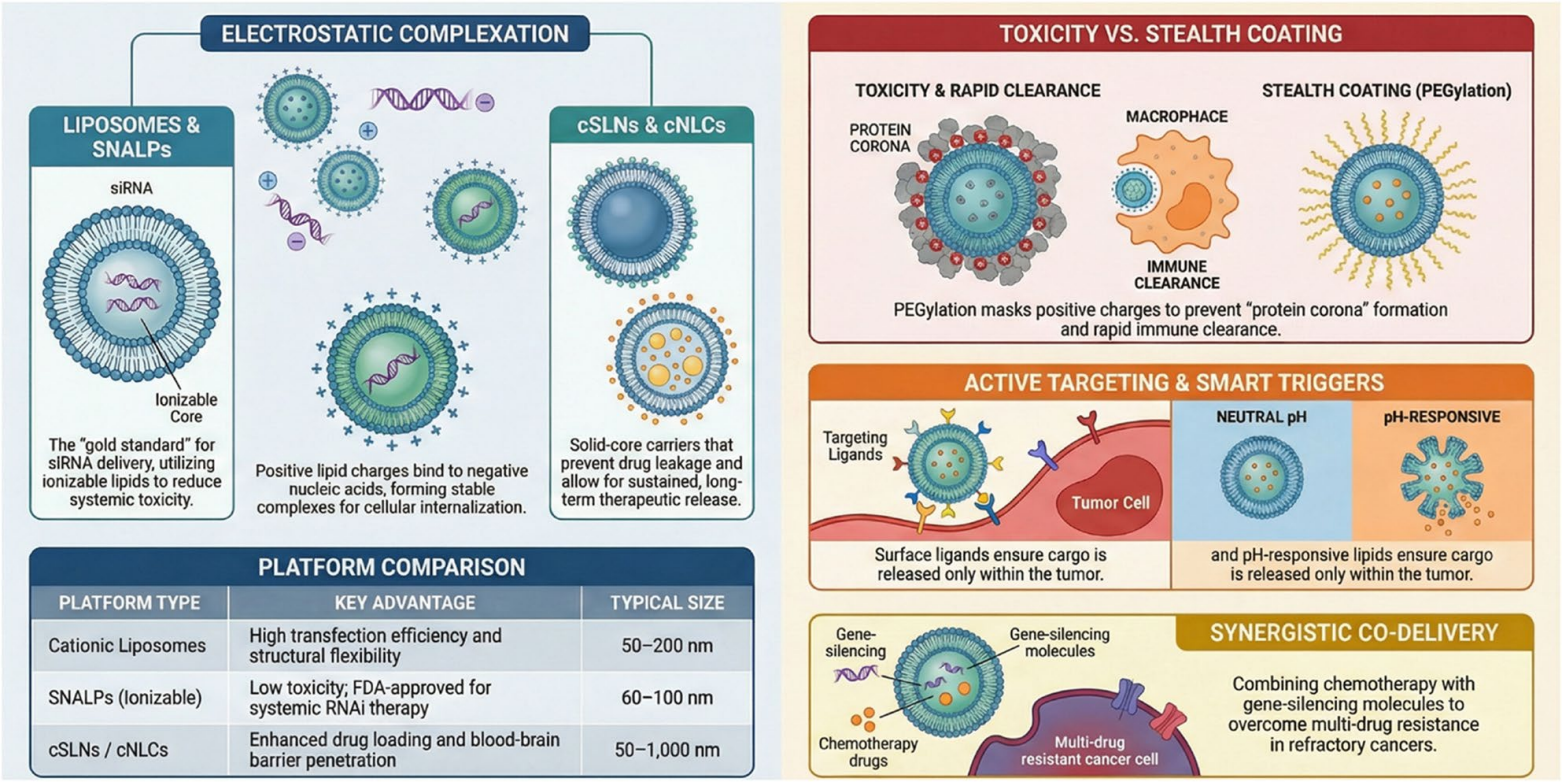
Overview of cationic-lipid based nanoparticle platforms and strategies for enhanced cargo delivery. Electrostatic complexation between positively charged lipids and negative nucleic acids forms stable complexes such as Liposomes, SNALPs (utilizing ionizable lipids), and solid-core cSLNs/cNLCs for various therapeutic applications. A platform comparison summarizes the key advantages and typical size ranges (50–1,000 nm) of cationic liposomes, SNALPs, and cSLNs/cNLCs. Advanced functionalization strategies include stealth coating (PEGylation) to prevent protein corona formation and immune clearance, thereby reducing toxicity. Active targeting and smart triggers utilize surface ligands and pH-responsive lipids to ensure tumor-specific cargo release. Finally, the platform enables synergistic co-delivery of chemotherapy drugs and gene-silencing molecules to overcome multi-drug resistance in refractory cancers

Conclusively, cLNPs demonstrate significant promise in cancer research as delivery platforms for therapeutic nucleic acids and proteins. However, their clinical translation is hindered by several critical challenges, including complex tumor biology, limited penetration into dense solid tumors, and potential off-target effects. Primary hurdles involve inherent toxicity from high positive charges and rapid immune clearance due to the formation of a protein corona in the bloodstream. Current research efforts are focused on strategies to mitigate these issues, such as coating NPs with PEG to extend circulation time and developing “smart” stimuli-responsive NPs that release their payload only within the TME (Fig. 3). Future perspectives emphasize integrating these carriers with immunotherapies and other treatments to enhance efficacy and prevent metastasis, moving toward personalized precision medicine.

In summary, the developmental trajectory of cationic lipid nanomedicine reflects a logical progression from simple electrostatic complexation to sophisticated, stimuli-responsive architectures. This evolutionary perspective highlights that the future of cancer therapy lies in the harmony between innovative nanoparticle engineering and a deeper understanding of the biological barriers presented by the TME.

## Data Availability

All data generated or analyzed during this study are included in this article, and no datasets were generated or analyzed during the current study. All datasets are available from the corresponding author upon request.

## Abbreviations

AAV: Adeno-associated viruses
ACP: Anti-cancer peptide
CFNP: Cargo-free nanoparticle
cLNP: Cationic lipid-based nanoparticle
cNLC: Cationic nanostructured lipid carriers
cNP: Cationic nanoparticle
cSLN: Cationic solid lipid nanoparticle
DC-Cholesterol: 3β-(N-[N’,N’-dimethylaminoethane]-carbamoyl)cholesterol
DDAB: Dimethyldioctadecylammonium bromide
DOPE: 1,2-dioleoyl-l-a-glycero-3-phosphatidylethanolamine
DOSPA: 2,3 dioleyloxy-N-[2-(sperminecarboxamido)ethyl]-N, N-dimethyl-1 propanaminium
DOTAP: 1,2-dioleyl-3-trimethylammonium-propane
DOTMA: N(1-[2,3-dioleyloxy]propyl)-N, N,N-trimethylammonium chloride
EPR: Enhanced permeability and retention
INF: Interferon
LNP: Lipid nanoparticle
MDR: Multidrug resistance
MNP: Metal-based nanoparticle
NP: Nanoparticle
PEG: Polyethylene glycol
RISC: RNA-induced silencing complex
SLN: Solid lipid nanoparticle
shRNA: Short hairpin RNA
siRNA: Small interfering RNA
ROS: Reactive oxygen species
SNALP: Stable nucleic acid lipid particles
TME: Tumor microenvironment
TNF: Tumor necrosis factor
TRAIL: Tumor necrosis factor-related apoptosis-inducing ligand
